# Exploring pectinolytic yeast diversity: toward effective polygalacturonase producers for applications in wine-making

**DOI:** 10.1093/femsyr/foae033

**Published:** 2024-12-18

**Authors:** Mehmet Gazaloğlu, Carole Camarasa, Elke Nevoigt

**Affiliations:** School of Science, Constructor University, Campus Ring 1, 28759 Bremen, Germany; UMR SPO, Univ Montpellier, INRAE, Institut Agro, 34000 Montpellier, France; UMR SPO, Univ Montpellier, INRAE, Institut Agro, 34000 Montpellier, France; School of Science, Constructor University, Campus Ring 1, 28759 Bremen, Germany

**Keywords:** pectin, yeast, pectinolytic activity, polygalacturonase, pectinase, screening, wine-making

## Abstract

Pectinolytic enzymes secreted by yeasts have an untapped potential in industry, particularly in wine-making. This study addresses the limitations of the current screening methods in reliably predicting the capacity of pectinolytic yeast strains to secrete polygalacturonase (PGase) under industrial conditions, suggesting a novel screening approach. Using the context of wine-making as an example, a diverse collection of 512 yeast strains from 17 species was analysed for PGase secretion, a key enzyme in pectinolysis. The traditional halo assay on solid yeast–pepton–dextrose (YPD) medium revealed 118 strains from nine genera being PGase positive. Screening these strains by incubating them at 20°C on a solid synthetic grape juice medium containing polygalacturonic acid (PG) significantly reduced the number of promising strains to 35. They belong to five genera: *Kluyveromyces* sp., *Cryptococcus, Pichia, Torulaspora*, and *Rhodotorula*. Afterward, a newly developed pectin–iodine assay was used to precisely quantify the PGase activity of the best-performing strains in a liquid medium. Strains from *Kluyveromyces* and *Cryptococcus* sp. stood out regarding high pectinolytic activity. Our methodological advancements tailored to identify highly promising pectinolytic yeasts for industrial use open new avenues for wine-making and other industrial processes encompassing media rich in pectin and sugars.

## Teaser

This study introduces a novel screening approach to identify yeast strains with high pectinolytic activity, optimizing polygalacturonase production for improved applications in wine-making and other pectin-rich industries.

## Introduction

Pectin is a critical component of plant-based food materials, predominantly found in the middle lamella of primary cell walls in fruits like citrus, apples, and grapes. Its complex structure includes major types such as homogalacturonan—a linear polymer of d-galacturonic acid—and rhamnogalacturonan I and II, which are branched heteropolysaccharides with varied backbones and side-chain compositions containing monosaccharides like l-rhamnose, arabinose, and galactose. The variability in pectin’s esterification and methylation significantly influences its structural and functional properties, presenting challenges when processing pectin-rich plant materials (Harholt et al. 2010). In wine-making, pectin affects the wine’s clarity, taste, and fermentation process, often leading to issues such as haziness and altered mouthfeel (Vanitha and Khan 2019, Espejo 2021, Jones-Moore et al. 2022).

Effective and sustainable methods for pectin degradation are essential in the food and beverage industry, highlighting the importance of pectinolytic enzymes—a diverse family with unique modes of action (Bhat 2000, Gummadi and Panda 2003). These enzymes break down pectin in various ways and are crucial for maintaining the quality and sensory attributes of wine (Mojsov et al. 2015, Martínez-Lapuente et al. 2019). They are also applied in fruit juice clarification and biofuel production (Semenova et al. 2006, Amin et al. 2019).

Grape must, the initial substrate for wine-making, contains a diverse community of naturally occurring yeasts, including species with pectinolytic activities such as *Candida, Hanseniaspora*, and *Pichia*. These yeasts produce pectinolytic enzymes that degrade pectin, facilitating juice extraction and clarification during early fermentation stages (Fernández et al. 2000, Strauss et al. 2001, Merín et al. 2015). Merín et al. (2013) found that among 78 yeast isolates, only 9 exhibited pectinolytic capabilities—including *Aureobasidium pullulans, Saccharomyces cerevisiae*, and *Filobasidium capsuligenum*—suggesting that while these yeasts are present, they are not dominant in fermentation. Despite their low prevalence, their enzymatic activities can significantly influence fermentation and the sensory attributes of the final wine by contributing to the release of phenolic compounds and flavour precursors from grape skins, enhancing complexity and quality (Jolly et al. 2014, Aponte and Blaiotta 2016, Bagheri et al. 2016, Ciani et al. 2016).

Due to the limited abundance of naturally occurring pectinolytic yeasts in grape must, there is interest in identifying and utilizing yeast strains with high pectinolytic activity for industrial applications. Traditionally, commercial pectinase cocktails derived from fungal sources like *Aspergillus* and *Trichoderma* have been used in wine-making. However, these preparations have disadvantages, such as containing enzymatic activities detrimental to wine quality, including pectin methyl esterase (PME), polyphenol oxidases, and cinnamyl esterases (Dugelay et al. 1993, Donaghy and McKay 1994, Malviya et al. 2011). The presence of PME raises safety concerns due to potential increases in methanol concentration in wine (Revilla and González-SanJosé 1998, Vilanova et al. 2000). Additionally, the production and use of these fungal enzymes involve extra costs and regulatory complexities (Raman and Henning 2013).

Several studies have indicated the untapped potential of yeasts for producing pectinolytic enzymes without introducing undesirable activities (Alimardani-Theuil et al. 2011, Geralda da Silva et al. 2005). Notably, certain yeast strains, such as *S. cerevisiae* and *Kluyveromyces marxianus*, have been shown to produce pectinolytic enzymes while lacking harmful enzymes like PME, polyphenol oxidases, and cinnamyl esterases (Blanco et al. 1999, Alimardani-Theuil et al. 2011). By carefully selecting and utilizing these specific yeast strains, winemakers can control the enzymatic profile introduced during fermentation, ensuring only beneficial activities are present.

Integrating such pectinolytic yeasts into the wine fermentation process has emerged as an innovative approach (Rollero et al. 2018). This strategy not only eliminates the need for traditional enzyme mixtures that may introduce undesirable enzymes but has also been reported to enhance the taste and sensory attributes of the final product (Fernández et al. 2000, Samanta 2019, Varela 2016). Rollero et al. (2018) demonstrated that the use of specific pectinolytic yeast strains can improve aroma complexity and mouthfeel in wines by facilitating the release of bound flavour compounds during fermentation.

This selective approach ensures that only pectinolytic activities are introduced, mitigating risks associated with unwanted enzymes. Techniques such as enzymatic profiling and genetic screening allow for the identification of yeast strains that lack harmful activities, providing control over the fermentation process (Alimardani-Theuil et al. 2011). Therefore, the introduction of these carefully selected pectinolytic yeasts does not pose the same risks as fungal pectinase cocktails, as it avoids the inclusion of enzymes detrimental to wine quality. This method offers a controlled and efficient means of enhancing wine production without compromising safety or quality.

The advantages of pectinolytic yeasts have led to growing interest in identifying suitable strains, prompting extensive screenings (Alimardani-Theuil et al. 2011). Assays commonly used for quantifying pectinolytic activity range from qualitative halo assays on solid media to quantitative assays in liquid media based on the liberation of reducing sugars (Gonçalves et al. 2010, Saranraj and Naidu 2014). However, existing screening methods have limitations and do not reliably predict a strain’s performance in actual wine-making processes. For example, the halo assay on solid yeast–pepton–dextrose (YPD) medium faces sensitivity and quantitative accuracy issues due to uneven nutrient diffusion (Atlas 2010, Madigan et al. 2019). Scaling these assays from laboratory to industrial scales is challenging, as plate conditions do not replicate the complex dynamics of liquid cultures (Doran 2013). The DNS (3,5-dinitrosalicylic acid) assay, frequently used to quantify pectinases in liquid media, encounters limitations in industrial applications where the inherent presence of reducing sugars, including esterified/methylated galacturonic acids, can interfere with its accuracy (O’Neill et al. 2020). Research by Belda et al. (2016), Paup et al. (2022), Rollero et al. (2018), and Servili et al. (1992) highlights the inadequacy of existing methods in differentiating between yeasts with basic and highly efficient pectinolytic activity.

To address these limitations, this study tested two new approaches: the solid synthetic grape must (SGM) medium and the pectin–iodine assay. These methods were designed to improve the detection and quantification of pectinolytic activities under conditions mimicking industrial applications. They better assess the capacity of yeast strains to produce polygalacturonase (PGase) under conditions similar to those during wine-making. We used these assays to test our collection of 512 yeast strains to find promising candidates for wine-making and other industries that utilize pectin and reducing sugars as raw materials.

## Materials and methods

### Yeast strains and preculture conditions

The yeast strains employed in this study were obtained from multiple renowned institutions: Supagro & INRA (Montpellier, France), University College Cork (UCC) (Cork, Ireland), Constructor University (Bremen, Germany), and BCCM/MUCL Agro-food & Environmental Fungal Collection, UC Louvain (Louvain-la-Neuve, Belgium). These strains listed in Table S1 were primarily isolated from environments predominantly involved in wine-making and in producing other fermented food and beverages. The *S. cerevisiae* strains L2323 (PGase positive) and L2226 (PGase negative) were used as controls and have been generously provided by Lallemand, Quebec, Canada.

Yeast cells were taken from the frozen glycerol stocks (stored at −80°C) and streaked onto solid YPD medium. The plates were incubated at 30°C for 48 h to obtain individual colonies. An overnight preculture was prepared by inoculating 4 ml of YPD in a glass tube with a single colony and the preculture was incubated at 30°C on an orbital shaker set at a shaking frequency of 200 rpm.

### Growth media

YPD medium contained 1% (w/v) yeast extract, 2% (w/v) peptone, and 2% (w/v) glucose. To prepare solid YPD medium, 2% (w/v) agar was added.

SGM medium (pH 3.5) is a synthetic medium mimicking the composition of grape juice (Bely et al. 1990). SGM contains 200 g/l of sugar (100 g/l glucose and 100 g/l fructose), 6 g/l malic acid, 6 g/l citric acid, and key vitamins and trace elements. We used a variation of this medium based on the method of Rollero et al. (2015), with β-phytosterols (5 mg/l) to meet the lipid requirements of yeast cells during anaerobic growth. All trace elements, vitamins, nitrogen sources, and anaerobic factors were filtered through a 0.22-µm syringe filter before being added to the pasteurized synthetic medium (80°C, 15 min). The pH was adjusted to 3.5 after pasteurization using a predetermined volume of KOH, based on prior testing to ensure consistent pH adjustment.

Two distinct media were utilized to screen for PGase enzyme-producing yeasts on solid media: YPD + PG medium (pH 5.0) and SGM + PG medium (pH 3.5). YPD + PG medium was prepared with modifications to the method described by McKay (1990) and Masoud and Jespersen (2006). Its composition represents that of solid YPD medium but contains 2% (w/v) polygalacturonic acid (PG; Sigma Aldrich-CAS 25990–7, ≥90% enzymatic). The solid SGM + PG medium corresponded to the liquid SGM medium see above which was supplemented with 2% (w/v) PG and 3% agar (w/v).

### Semiquantitative PGase assay on solid media (halo assay)

PGase activity assays on solid media relied on PG breakdown, resulting in a clear halo formation (Zink and Chatterjee 1985, Masoud and Jespersen 2006). In the current study, a 4 ml precultivation culture was diluted with a 0.9% NaCl solution until an optical density of 0.2 at 600 nm (OD600) was reached. 5 µl of this diluted culture were spotted on the respective solid agar plates. Each strain was tested with two biological replicates and four technical replicates (four spots for each biological replicate). We gently removed the yeast biomass using sterile water after incubating the plates at 30°C for 3 days. To visualize areas where PG was broken down by the yeast, the plates were treated with 6 M HCl for 15 min. Clear zones, or ‘halos’, around the spots where the cells grew, indicated PGase activity. The diameters of the halos were measured to obtain a rough estimate of the yeast’s PGase activity. All halo assays were conducted in duplicates, and mean values are shown.

### Pectin–iodine assay for quantification of pectinolytic activity in liquid medium

For the assessment of the activity of pectinolytic enzymes secreted by a yeast culture growing liquid medium, 100 ml of YPD medium was inoculated with the overnight preculture adjusted to an optical density of 0.2 at 600 nm. This culture was incubated at 30°C with orbital shaking at 200 rpm. In order to induce the secretion of pectinolytic enzymes, the YPD medium contained 2% (w/v) PG, as suggested by Solís-Pereira et al. (1993). At given time points (indicated in the text), a 1 ml sample of the culture was harvested and centrifuged at 11 200 × *g* for 5 min and filtered via a 0.22-µm membrane filter. The supernatant was employed for the pectinolytic enzyme quantification assay.

The pectin–iodine microplate-based assay used to measure pectinolytic enzyme activity in liquid yeast cultures was adapted from the starch–iodine assay published by Xiao et al. (2006). A 16.0 g/l pectin solution (Sigma-Aldrich CAS: 9000695) was prepared in a 50 mM acetate buffer with a pH of 5.0. In each well of a 96-well plate, 40 µl of this pectin solution was mixed with 40 µl of fermentation supernatant. After incubating this mixture at 40°C for 6 h, the reaction was halted by adding 20 µl 1 N HCl. This choice of 40°C as the incubation temperature was inspired by a previous study which stated that 40°C is within the optimal range for most pectinases (Blanco et al. 1999). The incubation time (6 h) was chosen based on preliminary optimization experiments, which indicated that this incubation time resulted in consistent enzyme activity measurements. We added Lugol solution with a slightly modified composition (5 mM I_2_ and 50 mM KI) to identify the remaining nondegraded pectin. The intensity of the brown colour resulting from pectin reacting with Lugol solution was measured at 580 nm using a microplate reader. The concentration of the remaining pectin was taken as a measure for the pectinase activity under the assumption that the latter is inversely proportional to the remaining pectin after the 6 h incubation period. To ensure accuracy, we included a calibration curve (different concentrations of a pectin standard solution) in each microplate.

### DNS-based assay of pectinolytic activity of a commercial enzyme mixture

The pectinase assay based on the DNS reagent, utilized to measure the release of reducing sugars through colorimetry in microtiter plates, was adapted from a method described by Gonalves et al. (2010). A calibration curve for the quantification of reducing sugars was prepared using a 2 g/l d-galacturonic acid solution, and absorbance was measured at 540 nm. The equation resulting from this calibration curve (Fig. S2) was used to determine the concentration of reducing sugars (d-galacturonic acid) based on absorbance values. A commercial pectinase preparation (Pectinex P2611, Sigma-Aldrich) and PG as a substrate were used to compare the DNS-based assay with the pectin–iodine assay.

## Results and discussion

### Initial screening of yeast strains for PGase activity on solid YPD medium

**Figure 1.**
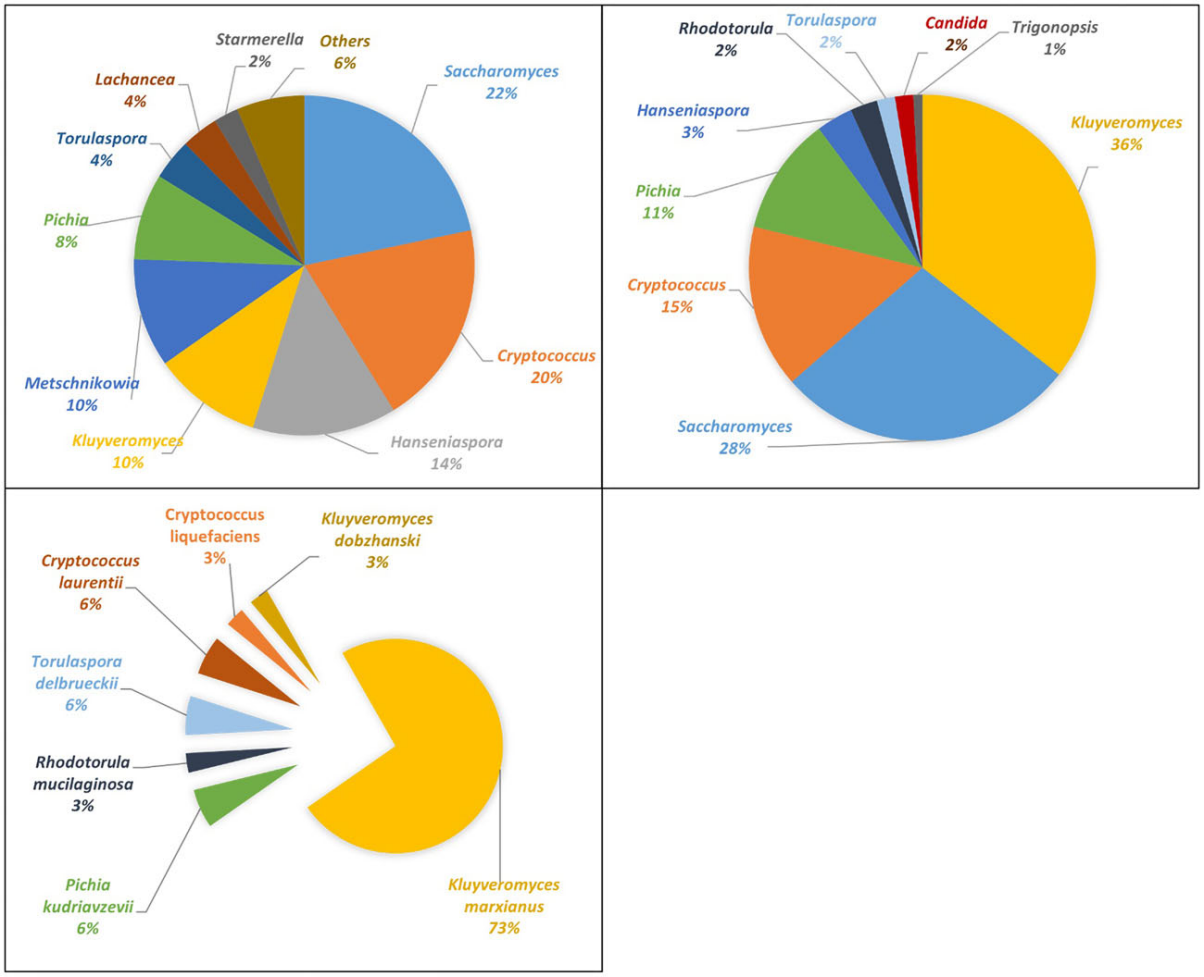
Distribution of tested yeast strains across yeast genera. (A) Collection of 512 yeast strains (from 17 genera) tested in this study. (B) Subcollection of 118 yeast strains, which exhibited detectable PGase activity on solid YPD + PG medium (30°C, pH 5.0). (C) Subcollection of (B): 34 yeast strains which retained PGase activity under conditions mimicking winemaking, i.e. solid SGM + PG medium (20°C, pH 3.5).

To identify potent pectinolytic yeast strains suitable for wine-making applications, we screened a diverse collection of 512 yeast strains representing 17 genera and over 50 species (Fig. 1A). This broad representation ensured a comprehensive evaluation of yeast variability relevant to agricultural and food applications. Many strains were isolated from grape juice and wine-associated environments, aligning with previous findings (Mateus et al. 2020 , Eder et al. 2017, Capozzi et al. 2015, Garofalo et al. 2016).

We evaluated PGase activity using a halo assay on PG-enriched YPD medium, as outlined in the section ‘Materials and methods’. This approach, used in previous studies (Martos et al. 2013, Masoud and Jespersen 2006), detects clear zones around yeast colonies due to PG hydrolysis by pectinolytic enzymes. *S. cerevisiae* L2323 (PGase-positive) and L2226 (PGase-deficient) strains were used as controls (Alimardani-Theuil et al. 2011, Meersman et al. 2017, Van Wyk and Divol 2010).

In contrast to previous studies, which often focused on yeast isolates from similar substrates and identified them only at the species level (Fernández et al. 2000, Merín et al. 2015, Belda et al. 2016), our approach was more comprehensive. We examined a broader spectrum of strains, including atypical grape juice isolates and unique *Cryptococcus* sp. (Fig. 1A). This diversity allowed for a more in-depth investigation into PGase production across various yeast strains and species, potentially expanding their application in wine-making and other sectors.

In the current work, yeast strains were considered PGase-positive if they produced a halo of comparable size (or larger) as the one produced by the PG-positive *S. cerevisiae* L2323 control. Notably, a substantial proportion of the tested *Kluyveromyces* strains (44 of 53 strains) exhibited the respective PGase activity, with *K. marxianus* emerging as the predominant species among PG-positive strains (Table S2, Fig. 1). In addition, 33 of 111 *S. cerevisiae* strains (30%) and 13 of 42 *Pichia* sp. strains (31%) were tested as PGase-positive. These values represent the percentage of PGase-positive strains within these species (Table S1), rather than the overall distribution shown in Fig. 1(B). Other PG-positive strains were identified from genera such as *Cryptococcus, Hanseniaspora*, and *Candida*, but the percentage was much lower. A significant observation from our study was the lack of PG activity in strains from species frequently associated with the wine environment, including 53 *Metschnikowia*, 18 *Lachancea*, and 12 *Starmerella* strains. Notably, Belda et al. (2016) reported PGase activity in a substantial proportion of tested *M. pulcherrima* isolates and *M. fructicola* strains. These contradicting results might have been caused by intraspecies phenotypic variability, the used growth conditions and medium composition, or the screening method itself. In fact, Belda et al. (2016) used Ruthenium Red staining and the stain has a broader affinity and can bind to other negatively charged compounds in addition to PG (Cook et al. 2013, Rashid et al. 2023). In agreement with the current study, other studies also did not detect pectinolytic activity in *Metschnikowia* strains (Pando Bedriñana et al. 2012, Charoenchai et al. 1997. , du Plessis et al. 2017). In previous studies, PGase activity was either absent or rare within the *Lachancea* genus, specifically in *L. thermotolerans* (Belda et al. 2016, Escribano-Viana et al. 2021, Vicente et al. 2021) and in the *Starmerella* genus, specifically in *S. bacillaris* (du Plessis et al. 2017, Englezos et al. 2015, Lemos et al. 2016). Thus, our observations supported the previously published data to a large part and also pointed to a low occurrence of pectinolytic species in the yeast community connected to wine-making environments. The major exception has been the *K. marxianus* species, which comprises many PGase-positive strains, even though there is a vast intraspecies diversity with regard to this property. Our comprehensive study confirms the view that enzymatic production in yeasts is not a characteristic inherent to any particular genus or species. Rather, it is a trait specific to individual strains. This insight into the diverse enzymatic capabilities across different yeast strains, including the well-studied species *S. cerevisiae*, underscores the importance of carefully selecting a suitable strain for a particular industrial context.

It is worth mentioning that the choice of the particular type of PG (Sigma Aldrich—25990–10 ≥ 90 enzymatic) as a critical inducer of pectinolytic enzyme production in both solid and liquid media was considered pivotal in our study. In fact, the use of PG in our solid media led to more distinct and measurable halos compared to pectin and other PG variations available on the market, making it the preferred choice for our assays.

### Evaluating PGase activity on solid medium using conditions relevant to wine-making

While the initial screening identified PGase activities in diverse yeast strains, the following section delves deeper into exploring their ability to also exhibit PGase activity under conditions relevant to wine-making. Based on the screening on solid YPD medium, 118 of our 512 strains were classified as PG-positive, spanning a range of nine different genera (Fig. 1B, Table S2). It is essential that the conditions under which our initial high-throughput prescreening was conducted (2 g/l glucose, pH 5.0, 30°C) significantly differ from those encountered in relevant industrial applications such as wine-making. Wine fermentations are typically conducted in media with higher sugar concentrations and a relatively low initial pH. Moreover, the temperature used for wine-making is significantly lower than 30°C (Ribéreau-Gayon et al. 2006). Notably, enzymatic activities, especially those related to pectin hydrolysis, are profoundly influenced by environmental conditions, notably by pH and temperature (Blanco et al. 1999, Haile and Ayele 2022).

In our pioneering approach to better adapt the screening for PGase secretion to wine-making conditions, we transformed the so-called SGM medium (Bely et al. 1990, Rollero et al. 2015), typically used in liquid form, into a solid SGM medium and added PG, thereby converting it into a medium suitable for a halo-based PGase screening assay targeted for wine-making conditions (see the section ‘Material and methods’). The cultivation of the cells on this medium was conducted at 20°C. This temperature is closer to the real wine-making conditions (compared to 30°C). The incubation time was chosen to be 3 days (72 h). The new assay method introduced in this study provides a more challenging medium composition that closely reflects wine-making conditions, offering a more practical platform for screening and predicting the strains’ potential performance in real wine fermentations. It might be worth mentioning that we also tested an incubation time of 5 days. However, strains that did not form halos during the first 3 days remained PGase inactive, even with prolonged incubation time. Still, strains being PGase positive after 3 days showed larger halos after 5 days.

When using our adapted plate assay (SGM + PG medium and 20°C incubation temperature), only 34 of the 118 strains initially identified as ‘PGase-positive’ still exhibited significant PGase activity. Notably, all of these strains showed smaller halo sizes compared to the assay initially used on the YPD medium and 30°C. Figure 1(C) shows how the strains can be allocated to seven different yeast species. Among them, *K. marxianus* stood out. This species accounted for 76.4% of all the strains showing PGase activity in the SGM + PG medium, a marked increase compared to the 35.6% observed in the YPD + PG medium. Notably, 26 of the 42 tested strains were still PG-positive when using the new assay (Table S2). One can conclude that *K. marxianus* is a yeast species with a high potential to identify suitable candidates for wine-making applications. Notably, two strains of *Torulaspora delbrueckii* also consistently displayed PGase activity under conditions simulating wine out of the 18 strains tested. Other species that retained PGase activity under these conditions included *Cryptococcus* sp. (3 out of 12), *Pichia* sp. (2 out of 13), and *Rhodotorula mucilaginosa* (3 out of 5). Remarkably, none of the tested *Saccharomyces* strains retained its pectinolytic activity when tested on a solid SGM + PG medium at 20°C. This activity loss may be due to the lower pH (3.5) and cooler temperature (20°C) in SGM, which can inhibit enzyme expression in *S. cerevisiae*. Furthermore, the nitrogen-poor composition of SGM, compared to nitrogen-rich YPD, is likely another limiting factor, as nitrogen is essential for enzyme production and gene expression in *S. cerevisiae* (Martínez-Moreno et al. 2012, Mendes-Ferreira et al. 2007, Godard et al. 2007, Contreras et al. 2011).

### Effects of assay conditions on halo size

**Figure 2.**
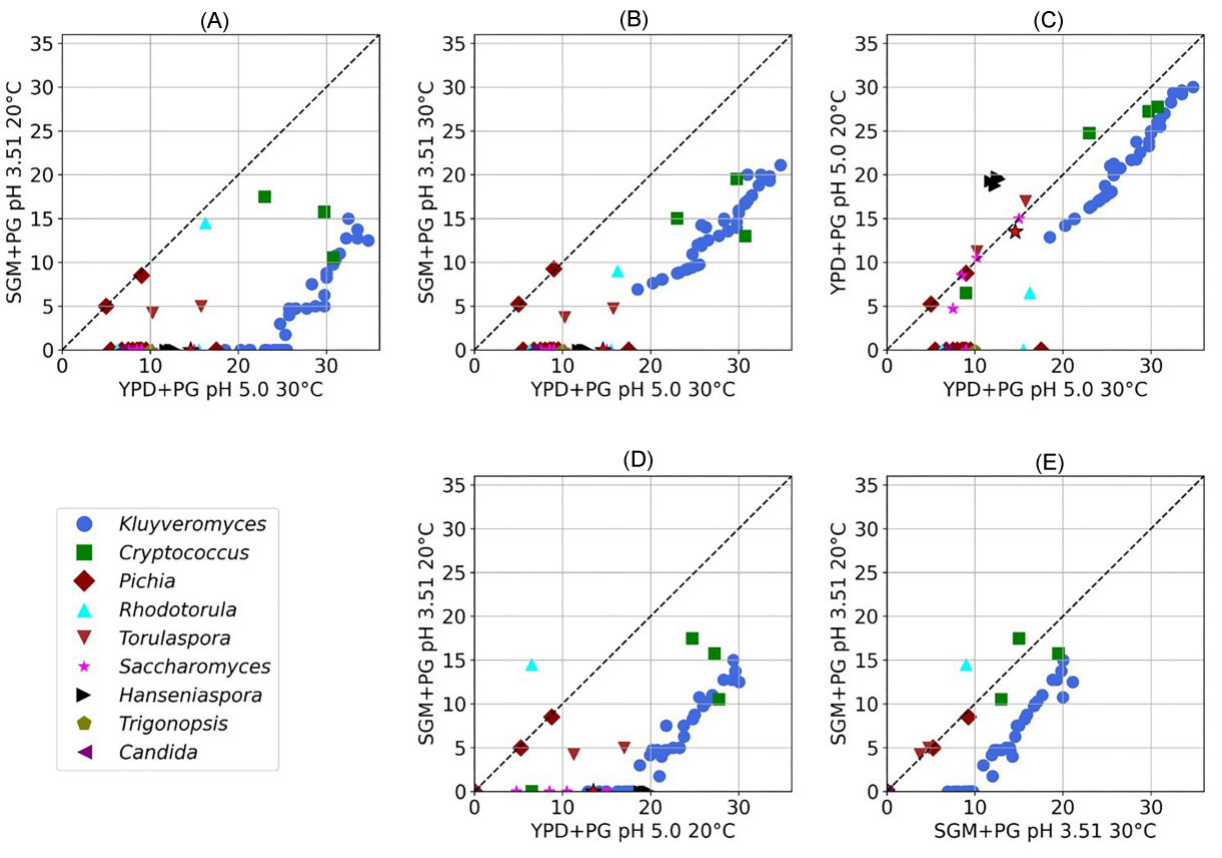
Comparative analysis of halo sizes (in mm) indicative of PGase activity produced by 77 yeast strains from nine genera on solid media when exposed to different conditions with regard to medium composition and incubation temperatures. The PGase-positive control strain *S. cerevisiae* L2323 is marked with star. The different scatter plots reveal how a change in the conditions affected the halo size. Each point on the plots corresponds to the mean value of two replicates of the halo size measured for a specific yeast strain, with distinct colours and shapes representing different genera. (YPD + PG: yeast extract peptone dextrose agar with 2% PG, SGM + PG; syntetic grape juice medium agar with 2% PG).

The next part of the study was conducted with a reduced number of strains (i.e. 77 out of the initially identified 118 PGase-positive strains). There were two reasons for this reduction: (i) strains without relevance for wine-making, such as *Cryptococcus* strains, were removed, and (ii) this part of the study was conducted in a different laboratory, and the COVID-19 pandemic resulted in severe logistical constraints exchanging yeast strains between countries. Despite the reduction in the number of strains, the chosen subset adequately represented the diversity of the original collection. As the next step, we spotted cells of the same yeast culture on both solid media (i.e. the YPD-PG medium at pH 5.0 and 30°C and our customized solid SMG + PG medium at pH 3.5 and 20°C) and compared the two halo sizes for each strain. The scatter plot (Fig. 2A) shows that halos became significantly smaller when the latter assay was used. Moreover, a large portion of the strains depicted as PGase positive on the YPD + PG medium did not produce any halo on the SMG + PG medium. However, Fig. 2(A) also reveals a correlation in PGase activities under the two tested conditions, even though this was only valid for certain *K. marxianu*s and *Cryptococcus* strains and required relatively high activity levels. Nevertheless, the high pectinolytic activity of the latter strains under the used experimental conditions indicates their potential for industrial applications.

To further dissect the influence of environmental conditions on PGase activity, we investigated how changes in medium composition and temperature affected the halo sizes of the tested 77 PG-positive strains. For this purpose, the same strains were analysed under two additional conditions: on the same SGM + PG medium but incubated at 30°C and on the common YPD + PG medium but at 20°C. Figure 2(B)–(E) represent key comparative analyses between the different media in order to demonstrate how changing particular conditions affected halo formation. For example, Fig. 2(B) demonstrates that maintaining the temperature at 30°C but switching from YPD + PG to SGM + PG medium already led to a significantly smaller halo for most strains. Notably, nearly half of the strains failed to produce any detectable halo, indicating a marked decrease in pectinolytic activity under the more challenging conditions of the SGM + PG medium. In fact, the grape-juice-like SGM medium has a higher sugar content (i.e. osmotic pressure), lower assimilable nitrogen, and a notably lower pH compared to YPD. Previous studies have shown that an increase in glucose concentration has a minimal impact on pectinolytic enzyme activity (Strauss et al. 2001, Serrat et al. 2004, Merín et al. 2015). Therefore, the pH differences might have played a pivotal role in influencing PGase activity in our modified assay conditions.

Interestingly, there was also a strong reduction in halo sizes when the temperature was changed from 30°C to 20°C. This can be seen for the YPD-PG medium in Fig. 2(C) and for the SMG-PG medium in Fig. 2(E). The comparison of the two graphs shows that the above temperature-induced reduction in PGase activity observed for many strains in the YPD medium was even more pronounced in the SGM + PG medium, with decreases in halo size ranging from 46% to 85%.

Interestingly, the significant decrease in PGase activity was not prevalent in all yeast strains investigated. For example, all tested *Pichia* spp. strains showed similar enzymatic activity when the temperature was decreased to 20°C. Surprisingly, the four analysed strains of *H. guillermondii* exhibited halos on YPD medium that had a larger studies involving another species of *Hanseniaspora*. Specifically, research on *Hanseniaspora uvarum* showed enhanced metabolic activity in cold storage conditions (Wang et al. 2019), indicating a similar trend among *Hanseniaspora* species. A comprehensive review by Albertin et al. (2016) highlighted the genetic diversity of *Hanseniaspora* species, which may contribute to their varied cold adaptation mechanisms, further supporting the enhanced metabolic activity observed in *H. uvarum* under cold conditions.

Generally, PGases, including those from *Saccharomyces fragilis* and *K. marxianus*, have been demonstrated to be most active above 30°C, with an optimal range of 40°C to 55°C and a preferred pH of 3.5–5.5 (Blanco et al. 1999). Nevertheless, the fact that the PGase activities of the different strains responded differently to variations in temperature or medium suggested that these changes are not only due to inherent properties of PGases of the various species but also to the impact of environmental factors.

Taken together, the results from the scatter plots in Fig. 2 (i) confirm our initial working hypothesis that the halo assay in YPD medium might not be the ideal screening condition to identify promising PGase-positive yeast candidates suitable for wine-making and (ii) show that both the temperature shift and the medium change significantly contributed to the different halo sizes obtained under laboratory and industrially relevant screening conditions. Altogether, the results of the scatter plots reveal a substantial metabolic diversity among the tested yeast strains. This diversity, rooted in evolutionary history, represents abundant opportunities for biotechnological applications.

### Development of an iodine-based method for quantifying pectinolytic activity in liquid media containing reducing sugars

In the quest to identify promising pectinolytic yeasts for wine-making, it is crucial to determine whether a pectinase is also produced in submerged cultures. This requires a method capable of quantifying pectinolytic activity in liquid media. As a side note, we hypothesize that evaluating pectinase activity in liquid media provides a more accurate reflection of a yeast strain’s ability to degrade pectin in industrial settings. This is in contrast to enzyme activity assays on solid media, where the secreted enzymes are mostly concentrated in a small area (the halo zone).

The conventional method for quantifying pectinolytic activity in a liquid medium involves the quantification of reducing sugars released during pectin hydrolysis. These sugars react with their free aldehyde or ketone groups using 3,5-dinitrosalicyclic acid (DNS) as a reagent (Merín et al. 2015, Poondla et al. 2015). Notably, this method falls short in several ways when applied to industrially relevant pectin-containing media such as grape juice. First, the hydrolysis of the complex pectin structure may release nonreducing sugars (Doco et al. 2001, Mudgil 2017), which are substantial components of pectin and not detectable by the DNS method (Miller 1959). There is a second obstacle that we need to consider here: synthetic grape juice contains reducing sugars (fructose and glucose) as well as free amino acids, and these compounds obviously affect the DNS assay’s accuracy in precisely quantifying pectinolytic activity (Teixeira et al. 2012).

To overcome the limitations, we developed an innovative alternative: the pectin–iodine assay. This method contrasts with the DNS assay by quantifying the remaining polymer rather than the sugars released during hydrolysis. The concept for the pectin–iodine assay was inspired by a previously published amylase activity assay that uses iodine to quantify starch (Fuwa 1954, Xiao et al. 2006), where starch breakdown is monitored through the colorimetric quantification of the starch–iodine complex, which forms a bluish-black colour measurable by absorbance at 580 nm.

Similarly, Lugol’s iodine reacts with pectin by forming complexes with its helical structure. The interaction of iodine with pectin is often compared to the well-known starch–iodine complex but is influenced by the degree of methoxylation in pectin. High methoxyl pectin (HMP) forms stronger and more stable complexes with iodine due to its more rigid helical structure, leading to a more intense and stable colour. In contrast, low methoxyl pectin (LMP), with fewer methoxy groups, forms less stable complexes with iodine, resulting in a lighter and less stable colour (Jaspers and Carstensen 2009, Finore et al. 2016).

This difference in the stability of colour formation between HMP and LMP may provide a means to observe different levels of enzyme activity depending on the methylation degree of the pectin used. As the pectinolytic enzymes break down these substrates, the rate of colour reduction could reflect the enzyme’s activity. Hence, this assay potentially allows for assessing enzyme effectiveness on both highly and lowly methylated pectin substrates (Mudarisova et al. 2020, 2023). While further validation is required, this approach offers a useful framework for comparing enzyme performance across various methylated pectin substrates. We adapted this approach to pectin, confirming that pectin also reacts with iodine to form a colourimetrically quantifiable complex, with the absorbance proportional to the pectin concentration (Fig. S1).

First, we used the novel pectin–iodine assay for quantifying the activity of a standard commercial pectinase (Pectinex P2611, Sigma-Aldrich) using PG as the substrate and compared the results with those obtained for the same solutions with the established DNS-based method (Fig. S2). This dual approach—measuring both the breakdown products of PG degradation (reducing sugars) and the degradation of the PG substrate itself—allowed us to draw a direct comparison between the two methods. The strong correlation between the obtained results (linear coefficient of determination = 0.9978; Fig. S3) confirmed that the pectin–iodine assay is as reliable and effective as the traditional DNS method in quantifying pectinolytic activity. Moreover, to validate the iodine-based assay’s robustness in diverse media conditions, we conducted additional experiments using phosphate buffer, SGM, and YPD media (pH 5.0). The assay demonstrated consistent pectinase activity readings across these media types, with the mean values showing only slight variations. The standard deviations remained within a statistically nonsignificant range of variability, as supported by ANOVA results (F = 0.561, *P* = .576; Table S3). This confirms low variability between replicates across different media.

We hypothesized that the newly developed pectin–iodine assay is also suitable for quantifying pectinolytic activity when using complex pectin rather than PG as a substrate. This approach will even provide a more holistic understanding of the pectinolytic activities present and crucial for applications in industrial settings where complex pectin structures are prevalent.

### Quantification of the pectinolytic activities of PGase-positive yeast strains in liquid medium using the new pectin–iodine assay

In order to apply the pectin–iodine assay for the quantification of pectinolytic activity of the best above-selected PGase-positive yeast strains, the initial plan was to cultivate the strains in a liquid version of the SGM + PG medium at 20°C. However, we faced significant challenges in achieving consistent yeast growth under these conditions. While yeast growth was feasible in the SGM medium alone, the addition of PG introduced complexities that hindered effective growth and, consequently, reliable enzymatic measurements. Therefore, we decided to grow the cells in a liquid YPD medium supplemented with PG.

The addition of PG in the cultivation medium facilitated pectinase production in submerged cultures, and the use of liquid YPD + PG medium ensured consistency with our solid media approach.

When quantifying the pectinolytic activity in the supernatants of our liquid yeast cultivations, it has to be emphasized that we added complex pectin (Sigma-Aldrich CAS: 9000695) before starting the actual enzymatic assay (see the section ‘Material and methods’). This approach allowed the assessment of the yeast strains’ ability to degrade real, complex carbohydrate substrates. The latter ability has been considered essential for the evaluation of their potential for practical applications, particularly wine fermentations.

**Figure 3.**
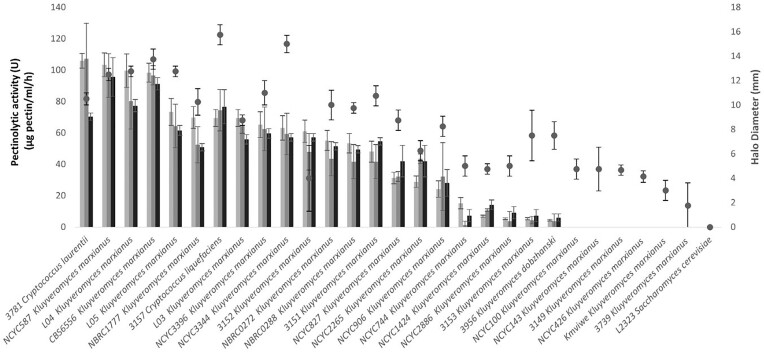
Pectinolytic activity of 28 yeast strains during cultivation in liquid YPD + PG medium after 24 h (white bars), 48 h (grey bars), and 72 h (black bars). The tested yeast strains primarily belong to the genera *Kluyveromyces* and *Cryptococcus* and represent those which produced a large halo on solid SGM + PG medium (incubation at 20°C) as visible by the circles (average values and standard deviations of two replicates). The *S. cerevisiae* strain L2323, used as a PGase-positive control strain in our initial screening on solid YPD + PG medium, has been included in the figure for comparison. The PGase activity of the supernatants was measured at 40°C of incubation with 16 g/l pectin solution at pH 5.5.

We evaluated the pectinolytic activities of 29 (out of the 34) strains that had previously shown significant halo formation on SGM + PG plates (Fig. 1C). We specifically analysed 26* K. marxianus* strains and 2 *Cryptococcus sp*. strains by quantifying pectinolytic activity in the supernatants of cultivations in YPD + PG at 30°C after 24, 48, and 72 h of cultivation (Fig. 3).

Of the 29 tested strains, 22 exhibited detectable pectinolytic activity when assessed with the pectin–iodine method. However, six *Kluyveromyces* strains, which initially tested positive for PGase activity on SGM + PG solid media, failed to show measurable activity in the subsequent liquid media conditions. These specific *Kluyveromyces* strains were the ones with rather small halos (<5 mm) on SGM + PG agar plates (Fig. 3). Strains NBRC1777 and L03 *K. marxianus* exhibited significant pectinolytic activity in liquid media despite their modest halo sizes of 10.25 mm and 8.75 mm, respectively, in SGM + PG solid media assay. This suggests that these strains, while not the top performers on solid media with PG, have a noteworthy ability to secrete enzymes and may be more effective in degrading complex pectin (rather than PG) in the liquid environment.

The pectin–iodine assay established in the current work represents a significant enhancement in resolution for characterizing pectinolytic activities of different yeast strains. Recently, Erasmus and Divol (2022) have suggested an alternative method in which supernatants of fermenting yeasts growing in liquid media is spotted on PG agar plates. However, this hybrid method (liquid/solid medium) still suffers from the major disadvantage of using solid media that it remains rather a qualitative assay and does not allow precise quantification and comparison of the capacity of yeasts to produce pectinases. The weakness of the spot assay is illustrated by the fact the strain L03 did not differ from strain L04 in the spot assay. 30% higher pectinase production capacity in our pectin–iodine assay (Fig. 3). The enhanced resolution of our assay method thus uncovers more subtle phenotypic variations among yeast strains, invaluable for industrial applications. Such a comprehensive understanding of enzyme capabilities is crucial for process optimization and product quality enhancement in industrial settings.

The pectin–iodine assay results confirmed *K. marxianus* as the predominant species among the isolates with high PGase activities, with 20 out of the 26 tested strains showing detectable activity. However, considerable intraspecies variability was evident again. The volumetric pectinase activity among these strains varied, ranging from about 100 µg of hydrolyzed pectin/ml/h for *K. marxianus* NCYC587 to as low as 5 µg/ml/h for *K. marxianus* 3153. Such variability within a species is a well-documented phenomenon in yeasts and can be attributed to genetic differences, mutations, or epigenetic modifications impacting enzyme production and secretion (Kang et al. 2019, Yi and Alper 2022).

Interestingly, most strains showed peak pectinolytic activity early in the cultivation process, particularly at 24 and 48 h, as depicted in Fig. 3. This pattern is consistent with the high metabolic activity typical of yeasts during their exponential growth phase, leading to increased enzyme production and secretion. As nutrient levels decrease and cells transition into the stationary phase, there is a corresponding reduction in metabolic activities. Envisaging the idea of combining pectinolytic non-*Saccharomyces* yeasts with *S. cerevisiae* in sequential fermentations in future wine-making scenarios, the early secretion of pectinase offers strategic advantages (Clemente-Jimenez et al. 2005, Ciani et al. 2014).

In departing from the traditional halo assay on solid YPD + PG medium for quantifying pectinolytic activity, our study has introduced two innovative methods: the use of solidified SGM adapted for PGase screening, and the pectin–iodine assay for the determination of pectinolytic activity in supernatants of liquid cultures. These two approaches are anticipated to enhance our capability to identify yeast strains with high pectinolytic activity tailored to the specific demands of wine-making. These assays can be conducted in parallel to study different aspects of pectinase production but could possibly be combined in the future into a single screening method with even more reliable predictive outcomes. Notably, promising pectinolytic strains identified in the current study were already used in actual fermentations, starting with real grape juice. The findings seem to confirm the value of the newly developed methods for predicting the performance of a particular strain under wine-making conditions.

## Supplementary Material

foae033_Supplemental_File

## Data Availability

All data generated or analysed during this study are included in this published article and its supplementary information files.
